# Operative versus conservative management of distal humerus fractures in patients aged 65 years and older: a target-trial-informed retrospective cohort study of five-year mortality

**DOI:** 10.1007/s00402-026-06514-x

**Published:** 2026-09-23

**Authors:** Hamza Murad, Mohammad Shehadeh, Ismaeel Igbaria, Muhamad Kiwan Mahamid, Mustafa Yassin, Assil Mahamid

**Affiliations:** 1 Department of Orthopedic Surgery, HaSharon Hospital (affiliated with the Gray Faculty of Medical & Health Sciences, Tel Aviv University, Tel Aviv, Israel), Rabin Medical Center, Petah Tikva, Israel; 2 Department of information systems, University of Haifa, Haifa, Israel

**Keywords:** Distal humerus fracture, Elderly, Target trial framework, Conservative management, Operative fixation, All-cause mortality

## Abstract

**Introduction:**

Older patients with distal humerus fractures die more often after conservative than after operative treatment in unadjusted comparisons; whether this reflects treatment or the selection of healthier patients for surgery is unresolved. We estimated the association between operative versus conservative management and five-year all-cause mortality in patients aged ≥ 65 years.

**Materials and methods:**

Retrospective comparative cohort study at two tertiary trauma centres (2012–2025). Cases were ascertained by automated screening of codes and free text, then adjudication of all uncertain records against source notes and injury radiographs, masked to treatment and vital status. Patients were classified by management initiated within 14 days as operative (*n* = 75) or conservative (*n* = 78); vital status was registry-linked. The primary estimand was the five-year restricted mean survival time (RMST) difference; principal adjusted estimates were inverse-probability-weighted (IPW) RMST and survival differences, requiring no proportional-hazards assumption. Cox, window and landmark analyses are supportive.

**Results:**

Among 153 patients (52 deaths), conservatively managed patients were older (median 83 vs. 73 years, *p* < 0.001). Unadjusted five-year mortality was 41.9% versus 18.4%: RMST difference + 11.2 months (95% CI + 5.6 to + 16.8), log-rank *p* = 0.006. After weighting for age, sex, comorbidity and centre this shrank to + 3.4 months (95% CI − 1.5 to + 8.6; adjusted five-year survival difference + 1.4%, − 14.7 to + 18.5), and to + 1.0 month (− 2.9 to + 5.5) within the covariate-overlap cohort (65–84 years). Comorbidity (HR 1.35 per Charlson point) and age (HR 1.72 per decade) predicted mortality in both arms. Sensitivity analyses were concordant.

**Conclusions:**

The large unadjusted survival advantage of operative fixation shrank substantially after adjustment, and the remaining estimates are too imprecise to support any claim of survival benefit or harm. Because functional outcomes, the principal indication for surgery, were not measured, the study cannot inform individual treatment choice. Crude survival differences here should not be read as treatment effects.

**Level of evidence:**

Level III, Retrospective comparative cohort study.

**Supplementary Information:**

The online version contains supplementary material available at https://doi.org/10.1007/s00402-026-06514-x.

## Introduction

Distal humerus fractures in older adults are typical fragility injuries — a low-energy fall, osteoporotic bone, a patient whose physiology matters more than the fracture pattern [1–3] — and their incidence is projected to rise steeply in elderly women [4]. Mortality after these injuries approaches that reported after hip fracture [1, 5–7], and registry studies consistently show higher death rates after non-operative than after operative treatment [5, 8]. Those same studies, however, show that non-operatively managed patients are older and more comorbid — the survival gap may say more about who is selected for surgery than about what surgery does [5, 6, 8].

Whether that gap survives confounding control is only now beginning to be tested [9]. Systematic reviews identify the absence of any direct operative-versus-conservative comparison in this fracture as the central evidence gap [10, 11]: the comparative literature addresses surgical-versus-surgical questions [12, 13], the non-operative evidence rests on single-arm series [14–16] against a substantial operative complication burden [11, 17], and a randomised trial powered for mortality is unlikely ever to be conducted. The target trial framework offers a principled structure for such an observational comparison — pre-specifying eligibility, treatment strategies, time zero, follow-up and estimand makes structural biases explicit [18, 19] — although it cannot substitute for randomisation, and confounding by indication must still be addressed analytically.

We therefore conducted a retrospective comparative cohort study, structured by the target trial framework, to estimate the association between operative versus conservative management and five-year all-cause mortality in patients aged ≥ 65 years with a distal humerus fracture, using the restricted mean survival time as the primary estimand, with elbow-related reoperation as a secondary endpoint.

## Methods

### Design and data source

We conducted a retrospective comparative cohort study, structured by the target trial framework [18], using routinely collected data from two tertiary trauma centres within a single university-affiliated hospital network (Centre A and Centre B; names withheld for double-anonymous review), covering fractures recorded between 2012 and 2025. The study was approved by the institutional review board of the participating network (approval number withheld for double-anonymous review), which waived informed consent given the retrospective, de-identified design. Reporting follows the STROBE statement. The analytical framework — the 14-day treatment window, time zero at emergency-department presentation, the five-year RMST estimand, the covariate set, and an analysis restricted to the zone of covariate overlap — was derived from the dated protocol of a companion emulation in a different elbow fracture, finalised before the outcome analyses of the present study (Supplementary Material). This was not a prospectively registered protocol for the present cohort, and the specific 65–84-year overlap bounds were derived empirically from the present data (Supplementary Fig. S1).

### Case ascertainment and verification

**Fig. 1:**
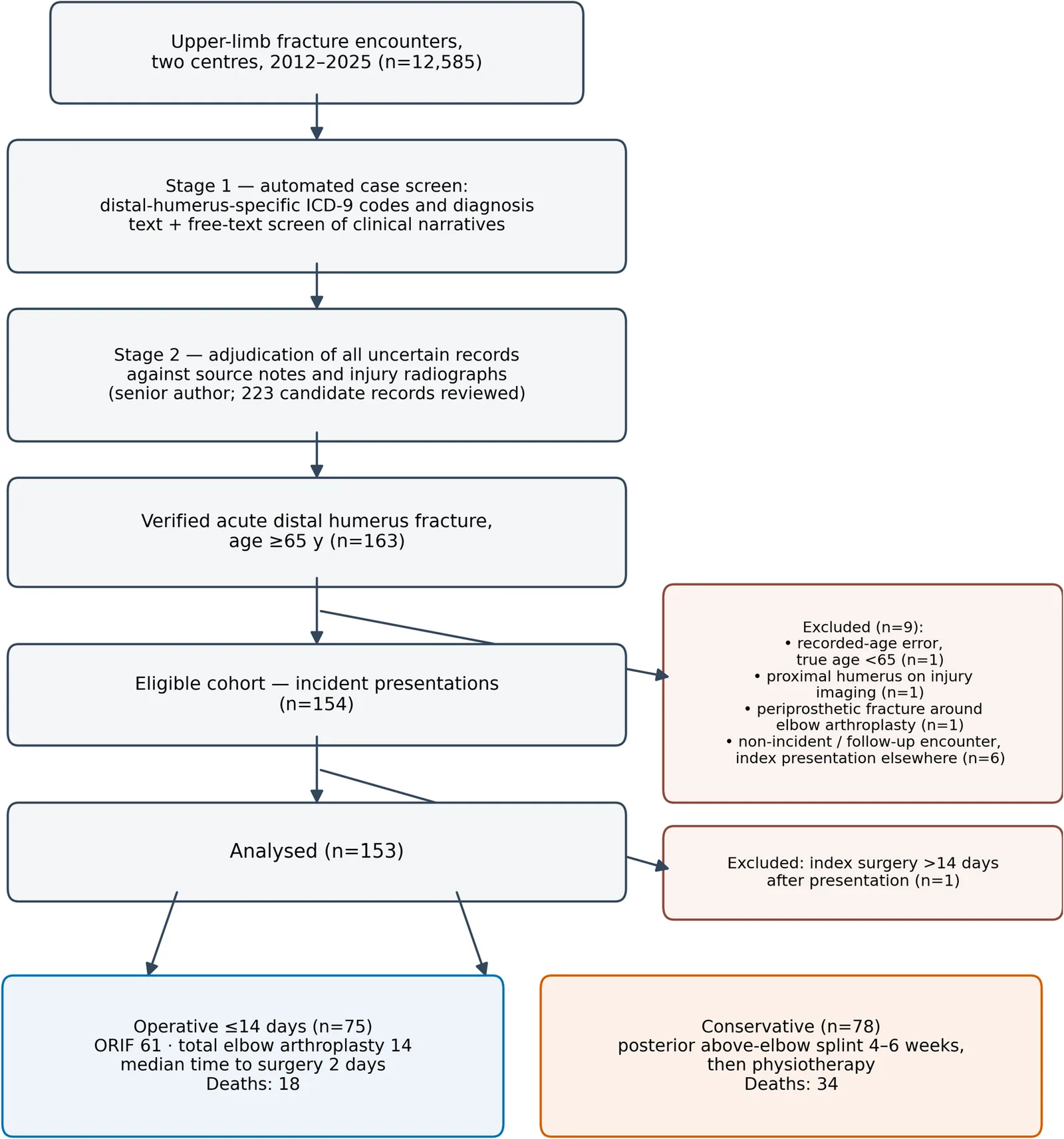
Study flow: two-stage case ascertainment with masked adjudication, eligibility, and allocation to operative and conservative strategies

Case ascertainment was two-stage (Fig. 1). First, all 12,585 upper-limb fracture encounters were screened automatically using distal-humerus-specific ICD-9 codes (812.4x/812.5x), diagnosis text, and a keyword screen of the clinical free-text narratives; ICD-9-CM remained the inpatient diagnostic coding standard at the study centres throughout 2012–2025. Second, every record whose fracture site could not be established with certainty from coding alone (*n* = 223) was adjudicated against the source clinical notes and the injury radiographs by the senior author. Adjudication was performed masked to treatment allocation and vital status: the surgery-date and vital-status fields were removed from the adjudication file before review, decisions were recorded against the source notes and the radiographs obtained at the time of injury, and the outcome fields were re-joined only after all decisions had been completed. Eligible patients were adults aged ≥ 65 years presenting with an acute native distal humerus fracture (extra-articular supracondylar and articular condylar/transcondylar/intercondylar patterns). Pathological fractures, periprosthetic fractures around an elbow arthroplasty, records with erroneous demographic data, and non-incident encounters were excluded, yielding 154 eligible patients (Fig. 1).

### Treatment strategies and protocols

The two treatment strategies were operative management — surgical fixation or arthroplasty of the index fracture initiated within 14 days of presentation — and conservative management (immobilisation without surgery). Conservative management comprised a posterior above-elbow splint for 4–6 weeks followed by routine physiotherapy. After both osteosynthesis and arthroplasty, patients received a posterior splint for 3 weeks, with physiotherapy instituted immediately and activity advanced as tolerated after splint removal; arthroplasty patients received a lifelong 4-kg lifting restriction. Non-operative care was selected through consultant-led assessment weighing fracture pattern, physiological reserve and comorbidity, premorbid function, and patient preference. The single patient whose index surgery occurred after 14 days was excluded from the primary comparison; after verification, no patient had undetermined surgical timing.

### Estimand

Time zero was the emergency-department presentation date. The primary comparison is an ‘as-treated-at-baseline’ contrast: the association of the management strategy actually initiated within 14 days of presentation with five-year all-cause mortality — an observational analogue of a per-protocol contrast, not an intention-to-treat estimand, since no baseline assignment exists in routine care. Because treatment classification uses post-baseline information, a landmark analysis is included (Statistical analysis).

### Outcomes and follow-up

The primary outcome was all-cause mortality. Vital status came from the hospital-network record system, which is synchronised with the national population registry through health-fund membership files and captures death dates irrespective of place of death; patients without a recorded death date were administratively censored on 30 November 2025. The primary estimand was the difference in restricted mean survival time (RMST) at five years, which requires no proportional-hazards assumption and reads directly as a difference in mean survival time [20]. The secondary outcome was elbow-related reoperation; coded one-year complication diagnoses are reported descriptively for both arms as lower bounds.

### Covariates and confounding

Pre-specified baseline covariates were age, sex, the Charlson comorbidity index (CCI) computed from coded comorbidities, and treating centre. Confounding by indication was addressed in three complementary ways. First, the principal adjusted treatment-effect estimates are inverse-probability-weighted (IPW) five-year RMST and survival differences, using a propensity score from a logistic model of treatment on age, sex, CCI and centre; balance diagnostics, weights and effective sample sizes are reported in Supplementary Table S1. Second, following the companion-protocol template, analyses were repeated within the empirical zone of covariate overlap (65–84 years; operative treatment was rare above 84: 7 of 43 patients), with positivity assessed from the propensity-score distributions (Supplementary Fig. S1; both arms represented in every propensity-score quintile, common support 0.13–0.82 covering 92% of patients). Third, multivariable and weighted Cox regressions are reported as supportive analyses. The E-value quantified the minimum strength of unmeasured confounding required to explain the adjusted association [21].

### Statistical analysis

Survival was described with Kaplan–Meier estimators and compared with the log-rank test; per-group RMST at five years is reported with bootstrap confidence intervals (1,200 replications). The principal adjusted estimates — the IPW-standardised five-year RMST difference and the adjusted five-year survival difference — were computed from weighted product-limit estimators, with the propensity model re-estimated within each of 1,000 bootstrap resamples. Cox hazard ratios (unadjusted, multivariable, overlap-restricted and IPW-weighted) are reported as supportive, time-averaged summaries because Schoenfeld-type tests indicated non-proportional hazards for treatment (*p* = 0.023) and age (*p* < 0.001; Supplementary Fig. S2); a quadratic age term improved model fit and is reported as a sensitivity analysis. Robustness was examined with 48-hour and 7-day treatment windows; a landmark analysis re-anchoring time zero at day 14 (patients not alive and under observation at day 14 excluded); reclassification of the single late-surgery patient as conservative; and a code-certain-subset analysis restricted to patients whose inclusion was determined by distal-humerus-specific diagnosis codes alone, involving no adjudication. Baseline characteristics were compared with the Mann–Whitney U or Fisher exact test. Two-sided *p* < 0.05 defined significance. The modeled covariates were complete for all patients; the CCI derives from coded diagnoses, so uncoded comorbidity scores zero. Analyses used Python 3.11 with the statsmodels library; the weighted models were verified against an independent implementation.

## Results

### Cohort and treatment

Of 163 verified acute distal humerus fractures in patients aged ≥ 65 years, 154 were eligible after nine exclusions, and 153 were analysed: 75 operative (61 ORIF, 14 total elbow arthroplasty; median time to surgery 2 days, IQR 1–3; 63% within 48 h, 92% within 7 days) and 78 conservative (Fig. 1). Conservatively managed patients were substantially older (median 83 vs. 73 years, *p* < 0.001); comorbidity was more prevalent in the conservative arm without reaching significance (CCI ≥ 1: 50.0% vs. 34.7%, *p* = 0.07; Table 1). Over a median follow-up of 4.1 years among survivors (maximum 12.6), 52 patients died (operative 18, conservative 34; after ORIF 13/61 and after arthroplasty 5/14, descriptive only). The design template and its implementation are summarised in Supplementary Table S2.

**Table 1:** Baseline characteristics and treatment characterisation by strategy

Characteristic	Operative ≤ 14 d (*n* = 75)	Conservative (*n* = 78)	*p*
Age (years), median (IQR)	73 (68–78)	83 (73–89)	< 0.001
Female, n (%)	56 (74.7)	61 (78.2)	0.704
Centre A, n (%)	62 (82.7)	59 (75.6)	0.324
Charlson index, median (IQR)	0 (0–1)	0 (0–2)	0.052
Charlson index ≥ 1, n (%)	26 (34.7)	39 (50.0)	0.072
Charlson index ≥ 2, n (%)	13 (17.3)	22 (28.2)	0.126
ORIF, n (%)	61 (81.3)	–	–
Total elbow arthroplasty, n (%)	14 (18.7)	–	–
Time to surgery (days), median (IQR; range)	2 (1–3; 0–12)	–	–
Follow-up, survivors (y), median (IQR)	4.3 (2.2–7.5)	3.8 (1.4–7.9)	0.907

p values from Mann–Whitney U or Fisher exact test

*IQR* interquartile range, *CCI* Charlson comorbidity index, *ORIF* open reduction and internal fixation, *TEA* total elbow arthroplasty

### Five-year mortality

**Fig. 2:**
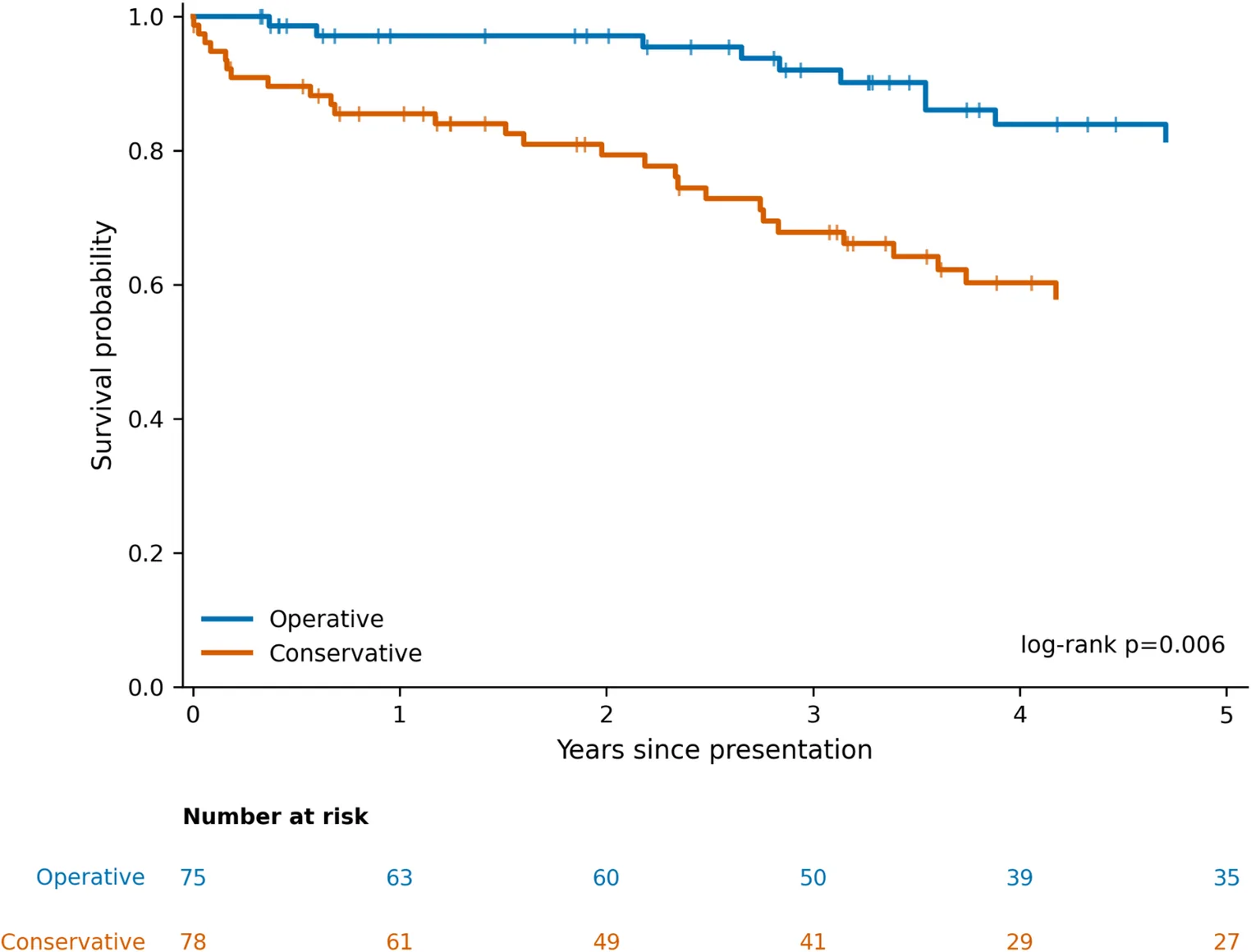
Unadjusted Kaplan–Meier survival by treatment strategy, full cohort, with numbers at risk

Without adjustment, conservative management was associated with markedly worse survival: five-year mortality 41.9% versus 18.4% (log-rank *p* = 0.006; Fig. 2), RMST 44.3 versus 55.5 months — a difference of + 11.2 months (95% CI + 5.6 to + 16.8) favouring surgery, crude HR 0.46 (95% CI 0.26–0.81). Two deaths occurred within the 14-day treatment window, both in conservatively managed patients (days 0 and 10); no patient documented as intended for surgery became unsuitable during the window.

**Fig. 3:**
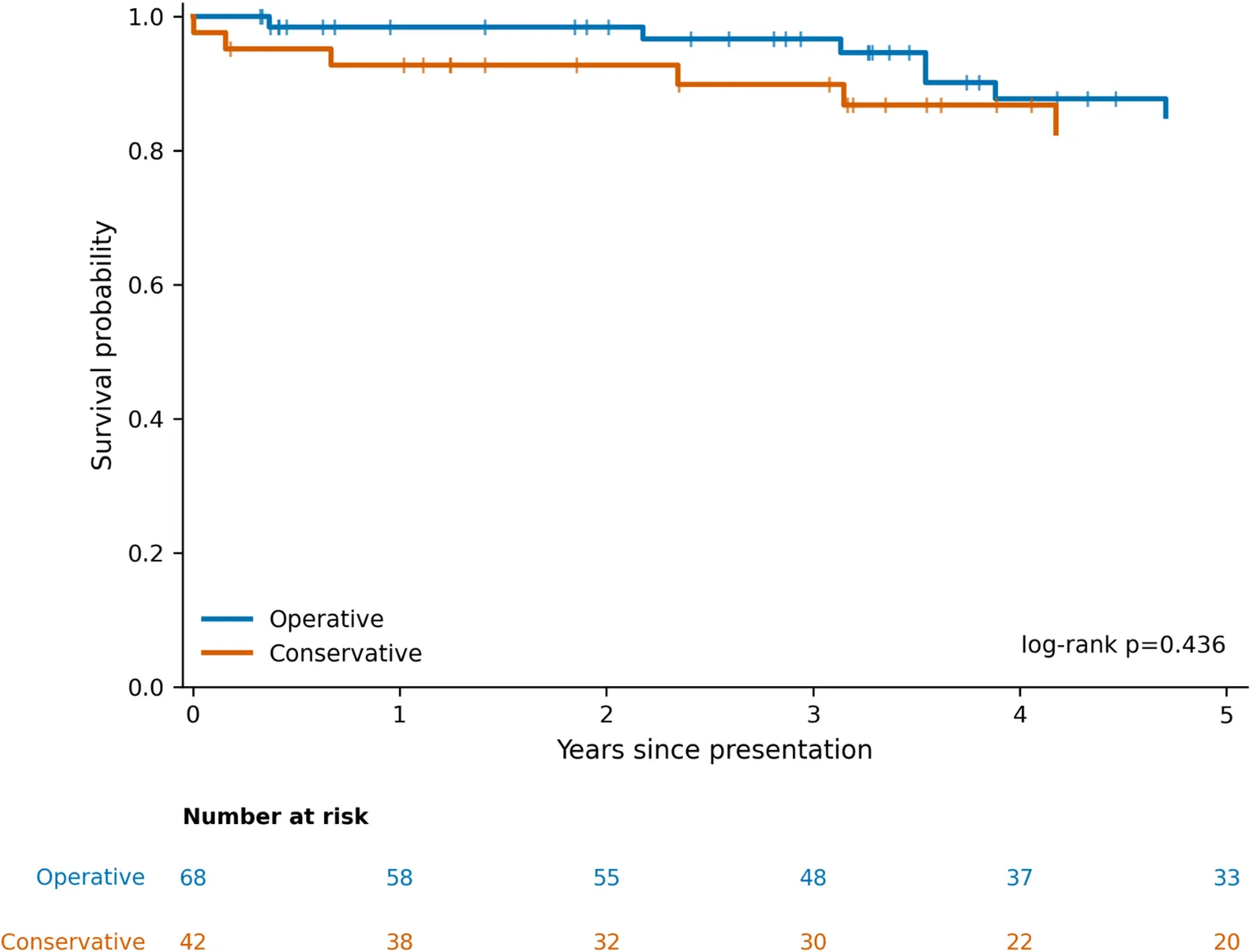
Kaplan–Meier survival in the covariate-overlap cohort (ages 65–84 years)

**Fig. 4:**
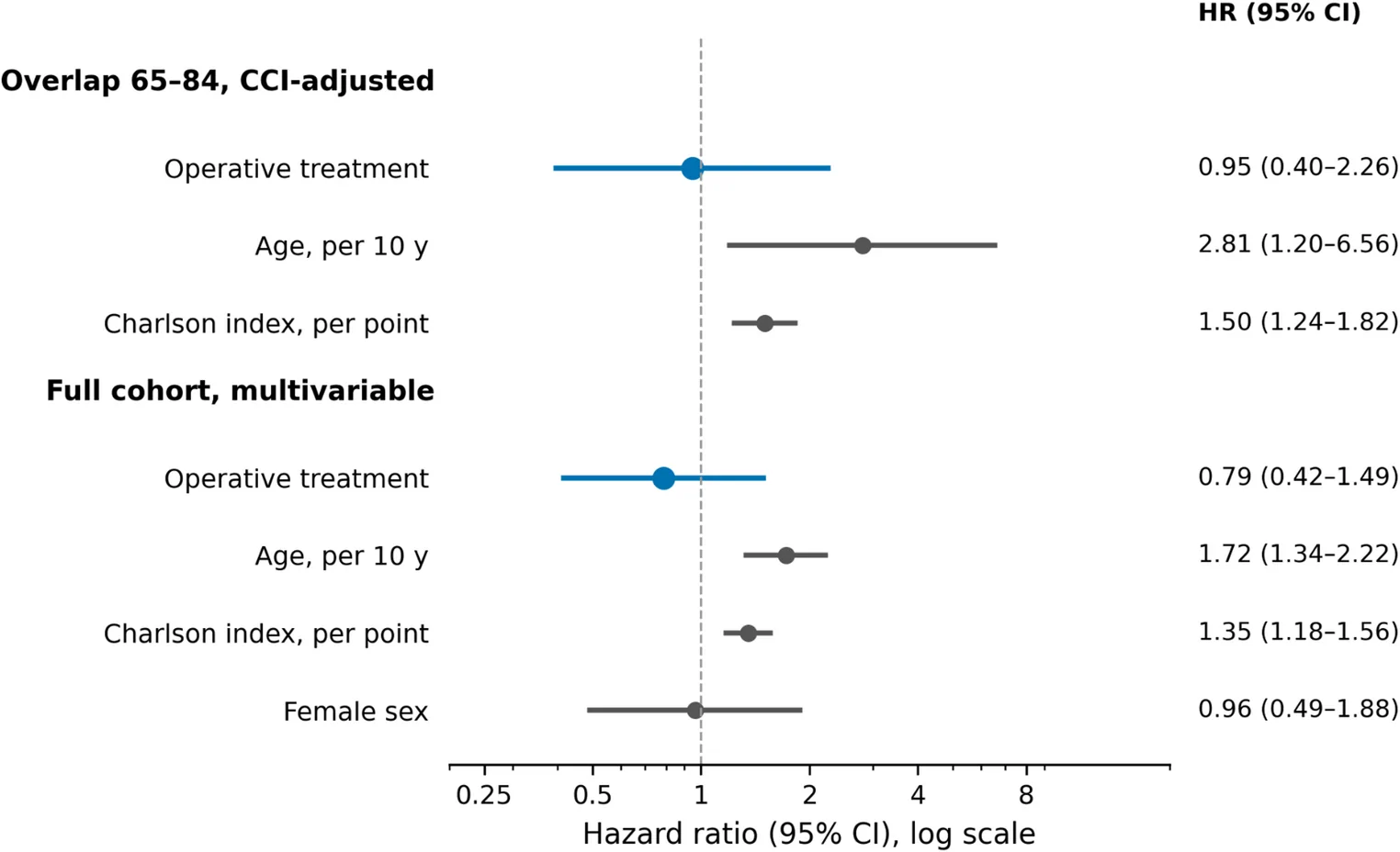
Supportive Cox hazard-ratio estimates for five-year all-cause mortality: the overlap-restricted adjusted model (age, sex, CCI) and the full-cohort multivariable model

Adjustment changed the picture. The IPW-adjusted RMST difference — the principal adjusted estimate — was + 3.4 months (95% CI − 1.5 to + 8.6; *p* = 0.20), with an adjusted five-year survival difference of + 1.4% (95% CI − 14.7 to + 18.5). Within the covariate-overlap cohort (65–84 years, *n* = 110; comorbidity still imbalanced: CCI ≥ 1 29.4% operative vs. 45.2% conservative), the adjusted RMST difference was + 1.0 month (95% CI − 2.9 to + 5.5; adjusted five-year survival difference − 3.8%, 95% CI − 17.6 to + 9.5), with unadjusted five-year mortality 14.8% versus 17.3% (log-rank *p* = 0.44; Fig. 3). Supportive Cox models agreed: overlap-restricted adjusted HR (age, sex and CCI) 0.95 (95% CI 0.40–2.26), full-cohort multivariable HR 0.79 (95% CI 0.42–1.49), IPW-weighted HR 1.08 (95% CI 0.60–1.75), and 1.00 (95% CI 0.53–1.90) under the better-fitting quadratic-age model (Table 2; Fig. 4). In both arms, mortality was predicted by comorbidity (CCI HR 1.35 per point, 95% CI 1.18–1.56) and age (HR 1.72 per decade, 95% CI 1.34–2.22; Table 3).

**Table 2:** Five-year mortality across analyses

Analysis	*n* (op/cons)	Deaths (op/cons)	5-y mortality, op vs. cons	Effect estimate (95% CI)	Supportive HR (95% CI)
Unadjusted, full cohort	153 (75/78)	18/34	18.4% vs. 41.9%	RMST diff + 11.2 (+ 5.6 to + 16.8)	0.46 (0.26–0.81)
IPW-adjusted, full cohort (principal)	153 (75/78)	18/34	25.8% vs. 27.2% (weighted)	RMST diff + 3.4 (− 1.5 to + 8.6); survival diff + 1.4% (− 14.7 to + 18.5)	1.08 (0.60–1.75)
Overlap 65–84, unadjusted	110 (68/42)	13/11	14.8% vs. 17.3%	RMST diff + 3.0 (− 2.0 to + 8.7)	0.73 (0.33–1.63)
Overlap 65–84, adjusted (principal)	110 (68/42)	13/11	–	IPW RMST diff + 1.0 (− 2.9 to + 5.5); survival diff − 3.8% (− 17.6 to + 9.5)	adjusted (age, sex, CCI) 0.95 (0.40–2.26)
Full cohort, multivariable Cox	153 (75/78)	18/34	–	–	0.79 (0.42–1.49); quadratic age 1.00 (0.53–1.90)
48-h window	125 (47/78)	10/34	20.3% vs. 41.9%	RMST diff + 10.1 (+ 3.3 to + 16.8)	0.42 (0.21–0.86); adj 0.76 (0.35–1.66)
7-day window	147 (69/78)	16/34	19.9% vs. 41.9%	RMST diff + 10.8 (+ 5.3 to + 16.8)	0.45 (0.25–0.81); adj 0.82 (0.42–1.60)
Landmark day 14	150 (75/75)	18/32	18.4% vs. 40.3%	RMST diff + 10.1 (+ 4.4 to + 15.8)	0.48 (0.27–0.86); adj 0.83 (0.43–1.58)
Late surgery as conservative	154 (75/79)	18/34	–	–	0.48 (0.27–0.85); adj 0.82 (0.43–1.55)
Code-certain subset (no adjudication)	84 (41/43)	7/16	12.8% vs. 41.4%	–	0.32 (0.13–0.78); adj 0.40 (0.14–1.13)

Principal adjusted estimates are the rows marked ‘(principal)’: inverse-probability-weighted (IPW) restricted mean survival time (RMST, months) and five-year survival differences (operative − conservative; bootstrap 95% CI, propensity model re-estimated per resample). Cox hazard ratios (HR) are supportive, time-averaged summaries. Adjusted models include age, sex and CCI (and centre for IPW)

### Sensitivity analyses

The pattern — a large unadjusted difference shrinking toward the null under adjustment — held throughout (Table 2): with 48-hour and 7-day treatment windows (crude HR 0.42 and 0.45; adjusted 0.76 and 0.82), in the 14-day landmark analysis (crude 0.48; adjusted 0.83), with the late-surgery patient reclassified as conservative, and in the code-certain subset requiring no adjudication (84 patients, 23 deaths; five-year mortality 12.8% vs. 41.4%; crude HR 0.32, 95% CI 0.13–0.78; adjusted 0.40, 95% CI 0.14–1.13). Descriptively, the crude association was strongest in the first year (HR 0.18, 95% CI 0.04–0.80) and attenuated thereafter (HR 0.40, 95% CI 0.17–0.94), consistent with early deaths among frail conservatively managed patients.

**Table 3:** Multivariable Cox regression for five-year all-cause mortality, full cohort (*n* = 153, 52 deaths) — supportive analysis (non-proportional hazards present for treatment and age; hazard ratios are time-averaged summaries)

Predictor	Adjusted HR	95% CI	*p*
Operative treatment	0.79	0.42–1.49	0.463
Age (per + 10 years)	1.72	1.34–2.22	< 0.001
Charlson index (per point)	1.35	1.18–1.56	< 0.001
Female sex	0.96	0.49–1.88	0.910

### Secondary endpoints

Elbow-related reoperation in the operative arm comprised two contracture/heterotopic-ossification releases and one probable nerve-decompression procedure (2.7–4.0%); no conservatively managed patient underwent delayed fixation or arthroplasty. Coded one-year complication diagnoses were rare in both arms (contracture: operative 4/75, conservative 0/78; nonunion, malunion, stiffness and surgical-site infection: none coded). These figures are lower bounds — coded capture understates complications, plausibly more so in conservative patients with less follow-up contact — and pain, instability and functional deterioration after conservative treatment were not systematically assessed, so the two strategies’ complication burdens cannot be compared from these data.

## Discussion

In this two-centre cohort of 153 older adults with a verified distal humerus fracture, conservatively managed patients died at more than twice the rate of operated patients over five years. That gap did not withstand confounding control: after weighting for age, sex, comorbidity and centre, the survival difference shrank from + 11.2 months to + 3.4 months (95% CI − 1.5 to + 8.6), and to + 1.0 month within the covariate-overlap cohort — estimates compatible with modest benefit, no effect, or harm. The same result emerged from every supportive model and every sensitivity analysis, including a landmark analysis addressing treatment-assignment timing and a code-certain subset untouched by case adjudication. Age and comorbidity, meanwhile, predicted death in both arms. The most defensible reading is the cautious one: the large crude survival difference chiefly reflects who was selected for surgery, and the independent association between treatment and survival remains uncertain.

This conclusion now has direct external support. Rice and colleagues, in a recent single-centre cohort of 82 older adults with isolated distal humerus fractures, likewise observed a large unadjusted mortality gap (two-year mortality 30.8% non-operative versus 9.3% operative) that did not survive adjustment: operative treatment was not independently associated with mortality, which the authors attributed to surgeon selection of healthier patients, with comorbidity burden and preinjury ambulatory status the dominant predictors [9]. Our study reaches the same conclusion over a five-year horizon with a direct operative-versus-conservative design, registry-linked mortality, and more extensive confounding analyses — overlap restriction, weighting, landmark and adjudication-free subsets. The convergence of two independent cohorts on the same attenuation pattern strengthens the case that registry-level survival differences in this fracture [5, 8] are largely artefacts of selection. The New York statewide analysis, which concluded that operative treatment remained protective after adjustment [5], could not adjust for the frailty, cognition and functional status that drive both treatment selection and death; our data, like Rice’s, suggest caution before reading such estimates causally — a divergence between unadjusted and adjusted or randomised estimates familiar from the proximal humerus [6, 22–24].

These findings carry limited implications for practice. This study measured no functional outcomes, and function — a usable hand, an elbow that permits independence — is the principal reason to operate on these fractures [10, 14–16]. A study of survival alone therefore cannot say whom to operate on. Nor does comorbidity’s dominance as a mortality predictor make it an operative selection criterion: comorbidity predicted death in both arms — it is prognostic, not shown to be predictive of treatment benefit. What the data do support is negative and useful: the expectation of a survival advantage should not drive the decision to operate, and the recumbency rationale — that early fixation permits mobilisation and thereby averts the fatal cascade of pneumonia, thromboembolism and deconditioning, as after hip fracture — while biologically plausible, found no detectable signature here once selection was accounted for. Treatment decisions should continue to rest on fracture characteristics, physiological reserve, baseline function, surgical risk and patient preference, informed by the functional literature; randomised or frailty-adjusted functional studies remain the way to settle the indication itself.

### Strengths and limitations

The study’s strengths are its verification and its estimand. Every uncertain record was adjudicated against source notes and injury radiographs, masked to treatment and vital status; mortality was registry-linked; the analytical framework was fixed in a dated companion protocol before these outcome analyses; and the principal adjusted estimate is an RMST difference — matching the primary estimand and requiring no proportional-hazards assumption, which the data demonstrably violate. Findings were triangulated across weighting, restriction, regression, landmark and adjudication-free analyses.

The limitations are those of routinely collected data. Treatment was not randomised, and the covariate set — age, sex, CCI, centre — cannot capture frailty, preinjury function, cognition, residential status, ASA class and anaesthetic risk, fracture severity and bone quality, associated injuries, rehabilitation potential, or patient preference; the E-value of 1.8 for the adjusted hazard ratio indicates that unmeasured confounding of moderate strength could fully explain, or reverse, the residual association. With 52 deaths, confidence intervals are wide, and a moderate survival effect in either direction cannot be excluded. Treatment classification was as-treated at baseline; early deaths could only be classified as conservative, a structural feature the landmark analysis addresses. The CCI derives from coded diagnoses; AO/OTA fracture classification and functional outcomes were unavailable. Registry linkage captures deaths irrespective of place of death for health-fund members, with residual misclassification possible for emigrants and non-members. The findings are two-centre and require external validation.

## Conclusion

In older adults with a distal humerus fracture, the large unadjusted five-year survival advantage of operative fixation shrank substantially after adjustment for age, sex, comorbidity and centre (IPW-adjusted RMST difference + 3.4 months, 95% CI − 1.5 to + 8.6), and the independent association between treatment strategy and survival remains uncertain: these data support neither a survival benefit nor a survival harm of operative management. Age and comorbidity predicted mortality in both arms. Because functional outcomes — the principal indication for surgery — were not measured, these findings cannot guide the choice of treatment for individual patients; their message is that crude survival differences in this population should not be interpreted as treatment effects, and that treatment decisions should continue to rest on fracture characteristics, physiological reserve, baseline function, surgical risk and patient preference.

## Supplementary Information

Below is the link to the electronic supplementary material.

Supplementary Fig. S1. Propensity-score distributions by treatment arm with the common-support region marked.

Supplementary Material 1

Supplementary Fig. S2. Complementary log-log survival plot; non-parallelism indicates non-proportional hazards, supporting the RMST as the primary effect measure.

Supplementary Material 2

Supplementary Material 3

## Data Availability

The datasets generated and/or analyzed during the current study are available from the corresponding author on reasonable request, subject to institutional data-governance approval.
