# Supplementary material for: Operative versus conservative management of distal humerus fractures in patients aged 65 years and older: a target-trial-informed retrospective cohort study of five-year mortality
Source: Arch Orthop Trauma Surg. 2026 Sep 23;146(1):344. doi: 10.1007/s00402-026-06514-x (PMC13601166; doi:10.1007/s00402-026-06514-x)
Supplement: Supplementary file 1 [file 402_2026_6514_MOESM3_ESM.docx]

**Supplementary Material**

# **Supplementary Table S1. Propensity-score model and inverse-probability-weighting diagnostics**

Propensity score: logistic regression of operative treatment on age, sex, Charlson comorbidity index (CCI) and treating centre (n=153). Logit coefficients: intercept 8.61; age (per 10 years) −1.20; female 0.11; CCI (per point) −0.01; Centre A 0.78. Conditional on the other covariates, treatment selection was driven almost entirely by age and centre; the CCI contributed essentially nothing to treatment prediction, although it strongly predicted mortality in the outcome models — the signature of confounding by indication operating through age.

*Standardised mean differences (SMD) before and after inverse-probability weighting.*

| **Covariate** | **SMD before weighting** | **SMD after weighting** |
| --- | --- | --- |
| Age | −0.95 | 0.00 |
| Female sex | −0.08 | 0.01 |
| Charlson comorbidity index | −0.22 | −0.03 |
| Treating centre (Centre A) | 0.17 | −0.03 |

Weights: median 1.57 (IQR 1.30–2.12), maximum 7.9. Effective weighted sample sizes: operative 54 (from 75), conservative 59 (from 78). Common-support region of the propensity score: 0.13–0.82, containing 92% of patients.

*Patients and deaths by propensity-score quintile and treatment arm.*

| **Quintile (PS range)** | **Operative n (deaths)** | **Conservative n (deaths)** | **Comment** |
| --- | --- | --- | --- |
| Q1 (0.01–0.26) | 4 (3) | 27 (19) | both arms represented |
| Q2 (0.26–0.40) | 11 (5) | 19 (9) |  |
| Q3 (0.41–0.60) | 15 (2) | 16 (4) |  |
| Q4 (0.60–0.71) | 24 (4) | 6 (1) |  |
| Q5 (0.71–0.85) | 21 (4) | 10 (1) |  |

# **Supplementary figure legends**

**Supplementary Fig. S1.** Propensity-score distributions by treatment arm, with the common-support region marked. Both arms are represented in every propensity-score quintile.

**Supplementary Fig. S2.** Complementary log-log survival plot by treatment arm. Non-parallelism indicates non-proportional hazards for treatment (Schoenfeld-type test p=0.023) and age (p<0.001), supporting the restricted mean survival time as the primary effect measure.

# **Supplementary Table S2. Design template and its implementation**

*Supplementary Table S2. Design template (from the companion-study protocol) and its implementation in the present study.*

| **Element** | **Design template** | **Implementation in this study** |
| --- | --- | --- |
| Eligibility | Adults ≥65 y with an acute native distal humerus fracture | Two-stage ascertainment: automated ICD-9/text screen of 12,585 encounters + masked adjudication of uncertain records against source notes and injury radiographs; pathological, periprosthetic and non-incident encounters excluded |
| Treatment strategies | Operative fixation or arthroplasty ≤14 d vs conservative management | Operative n=75 (ORIF 61, TEA 14); conservative n=78 (posterior splint 4–6 weeks, then physiotherapy); one late surgery (day 17) excluded |
| Assignment | Randomisation (hypothetical) | As-treated at baseline; confounding addressed by IPW (principal), overlap restriction, multivariable Cox (supportive) |
| Time zero | Emergency-department presentation | Same; landmark analysis at day 14 addresses treatment-assignment timing |
| Outcome | 5-year all-cause mortality | Registry-linked vital status via health-fund membership files; administrative censoring 30 Nov 2025 |
| Estimand | RMST difference at 5 years (primary) | Same; principal adjusted estimate = IPW-standardised RMST difference (bootstrap CI); hazard ratios supportive (non-proportional hazards present) |
